# Supplementary material for: A Candida parapsilosis Overexpression Collection Reveals Genes Required for Pathogenesis
Source: J Fungi (Basel). 2021 Jan 29;7(2):97. doi: 10.3390/jof7020097 (PMC7911391; doi:10.3390/jof7020097)
Supplement: Supplementary file 1 [file jof-07-00097-s001.zip › Supplementary_data.docx]

**Supplementary Information**

**Table S1: Strains and primers used in this study.**

Table contains the genotypes of the applied strains and their references. Oligonucleotides for Gateway^TM^ cloning technology (with attB sites, BAR codes and gene specific sequences) primers for real-time, colony PCR and Southern blot experiments.

**Table S2: Applied stressors and their concentrations in solid YPD media for spot assay analysis.**

**Table S3: The list of the selected genes for OE analysis.**

The table lists the selected genes (and their ortholog’s functions) for OE experiments based on preliminary results [2,3,4] and some additional genes whose othologs have known virulence or stress tolerance related functions in *C. albicans* or in *S. cerevisiae*. The table also indicates if a KO of a given gene is available and if the OE mutant showed any altered phenotype compared to the control strain in this study.

**Table S4: The results of pseudohypha formation experiments.**

The table details the results of the pseudohypha analysis of the overexpression mutant strains carried out with Amnis® FlowSight® flow cytometer. Cells were cultivated in DMEM containing 10% (*v/v*) HI FBS or in YPD for 48 hours at 37 °C in the presence of 5% (*v/v*) CO_2_. The shape of the non-aggregated cells was characterized as yeast or elongated.

**Table S5: The results of the antifungal susceptibility analysis.**

The table details the results of the antifungal susceptibility test of the overexpression mutant strains exposed to 3 echinocandin drugs (anidulafungin, caspofungin, micafungin). Final drug concentrations were from 8 µg/mL to 0.0156 µg/mL with 3 × 10^3^ fungal cells in 200 µL final volume of RPMI-MOPS. Cells were incubated at 30 °C without shaking and monitored after 24 and 48 hours. MIC values for the applied drugs were defined as the lowest concentrations that resulted in at least 50% growth reduction. In addition to mCherry^OE^, CPRI and CLIB214 *C. parapsilosis* strains, a *C. krusei* strain with known antifungal susceptibility was also included as a control.

**Table S6: The summarized results of the OE mutant collection analysis.**

The table presents mutant strains with altered phenotypes and the comparison with former results of KO or OE mutant library analysis regarding *C. parapsilosis*, *C. albicans* and *S. cerevisiae*.

**Supplementary data**

**Figure S1:** **The schematic figure of the OE vector.**

The figure shows the structure of the expression vector used for mutant generation. Figure was modified based on the work of Németh and colleagues [48].

**Expression vector**

**7045 bp + ORF**

**Figure S2: Representative figures of** **the methods for verification of the generated OE mutant strains.**

**A)** Figure shows the schematic structure of the overexpression construction integrated into the CpNEUT5L region. Arrows show the sites of the gene specific real-time and the universal primers for rapid screen of correct integration. T: terminator. Figure was modified based on the work of Németh and colleagues [48]. **B)** Representative gel electrophoresis figure shows the verification of selected OE strains by colony PCR. Samples: 1. mCherry^OE^ – 1900 bp; 3. CPAR2_108840^OE^ – 1921 bp; 5. CPAR2_109520^OE^ – 1722 bp; 7. CPAR2_302400^OE^ – 1402 bp; 9. CPAR2_500180^OE^ – 1902 bp; 2., 4., 6., 8., 10. samples are no-template controls. **C)** The schematic figure represents the linearized overexpression plasmid integrated into the CpNEUT5L region and highlights the features of the Southern blot analysis. Figure was modified based on the work of Németh and colleagues [48]. **D)** Representative figure of the control, parental and OE mutant strains verified by Southern blot analysis. Samples: 1. CLIB214; 2. CPRI; 3. CPL2; 4. mCherry^OE^; 5. CPAR2_108840^OE^; 6. CPAR2_109520^OE^; 7. CPAR2_302400^OE^; 8. CPAR2_500180^OE^.

x bp

**PCR check product** (x+1171 bp)

CpN5L Up

WT allele

CPAR2_303830

ReTi_REV

ReTi_FOR

OE allele

CpN5LUp

1171 bp

CpN5LcheckREV

CpN5L Down

CpNEUT5L

(Up+Down)

CpN5LDo

_Cm_LEU2

P_Ca_TDH3

CpORF

TAG

T

CpN5LUp

CpN5LDo

CPAR2_303820

**A)**

**B)**

**M 1 2 3 4 5 6 7 8 9 10**


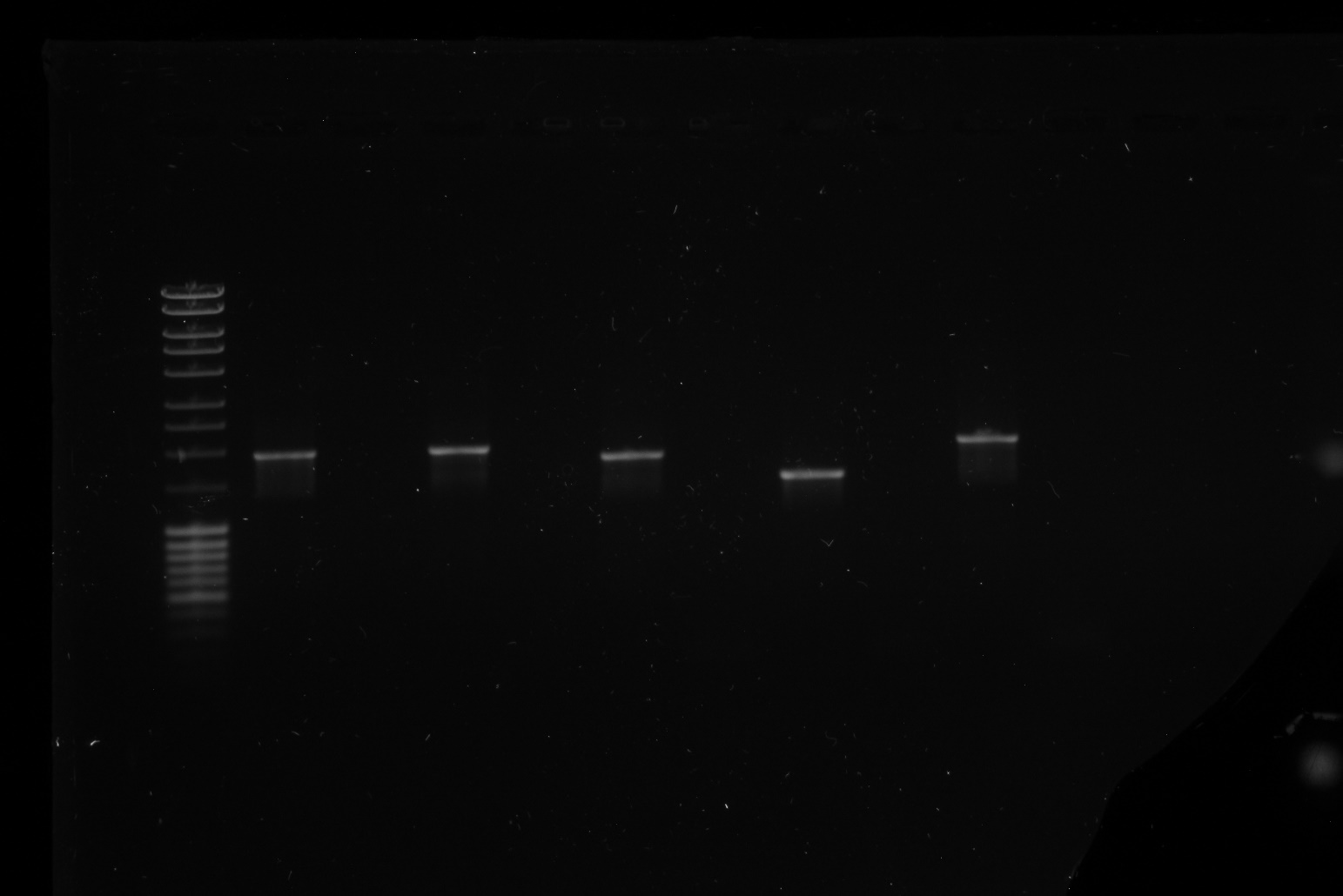


**2500 bp**

**2000 bp**

**1500 bp**

**C)**

*EcoRI* restriction sites:

DIG-labelled probe:

*EcoRI*

*EcoRI*

*EcoRI*

CpN5L Up

WT allele

CPAR2_303830

OE allele

CpN5LUp

CpN5L Down

CpNEUT5L

(Up+Down)

CpN5LDo

_Cm_LEU2

P_Ca_TDH3

CpORF

TAG

T

CpN5LUp

CpN5LDo

CPAR2_303820

7821 bp

5514 bp

5255 bp

*EcoRI*

*EcoRI*

*EcoRI*

**D)**


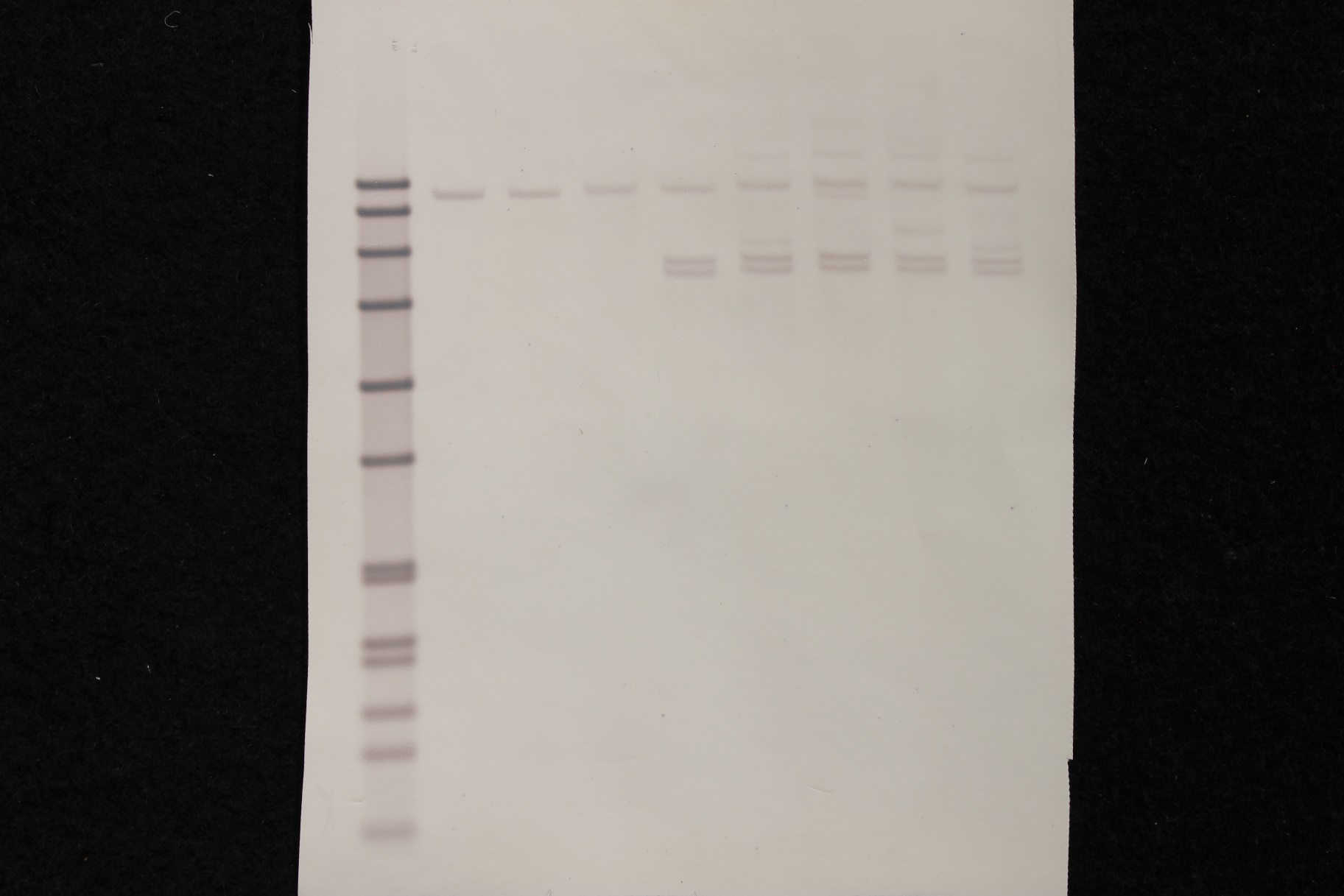


M 1 2 3 4 5 6 7 8

**8576 bp**

**7427 bp**

**6106 bp**

**4899 bp**

**7821 bp**

**5514 bp**

**5255 bp**

**Figure S3:** **Results of the growth kinetic measurements in complete media (YPD).**

Graphs show the growth kinetic analyses of the OE strains in YPD liquid media at 30 °C. N=3, with 3 technical parallels per strain. All the three control strains were tested.

**Figure S4:** **Results of the growth kinetic measurements in minimal media (YNB + 0.5% (*m/v*)** **glucose).**

Graphs show the growth kinetic analyses of the OE strains in YNB + 0.5% (*m/v*) glucose liquid media at 30 °C. N=3, with 3 technical parallels per strain. All the three control strains were tested.

**Figure S5:** **Results of the growth analyses on solid minimal media and with additional serum.**

Representative figures show the fitness of some OE mutant and the 3 control strains on solid minimal media (0.19% YNB + 0.5% (*m/v*) glucose with or without of 10% (*v/v*) FBS) at 30 or 37 °C after 48 h incubation.

**
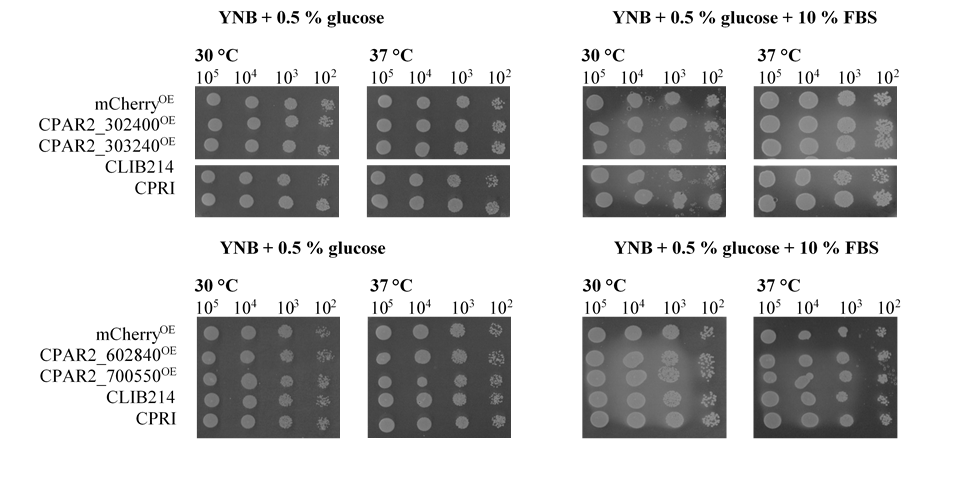
**

**Figure S6: Results of the growth analyses of the 3 control strains on solid media supplemented with different stress agents.**

Representative figures show the fitness of the 3 control strains on solid media supplemented some stressor agents at 30 or 37 °C after 48 h incubation.


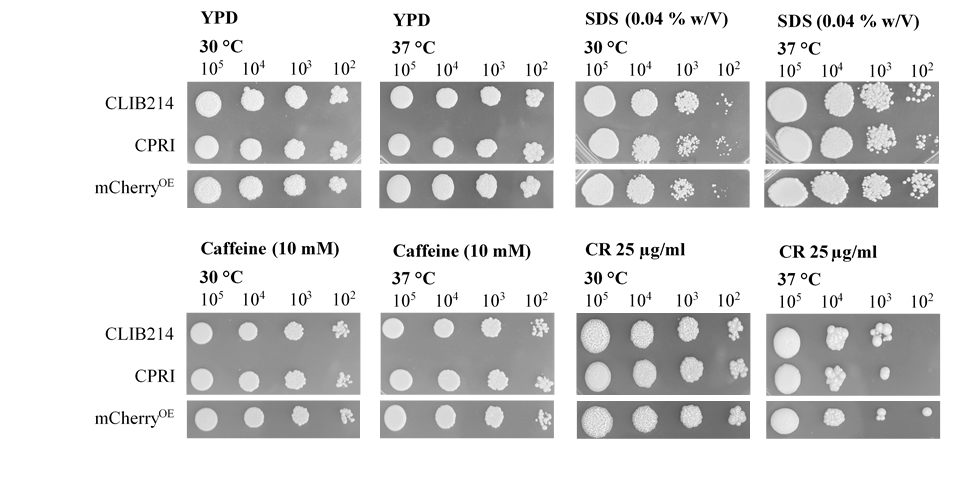


**Figure S7:** **Representative figures from pseudohypha analysis by Amnis® FlowSight® flow cytometer.**

**A)** Analysis of the CLIB214 strain in YPD and DMEM + 10% (*v/v*) FBS medium. **B)** Analysis of the CPAR2_302400^OE^ strain in YPD and DMEM + 10% (*v/v*) FBS medium. **C)** Representative light microscopic pictures of yeast, aggregated and elongated/filamentous cell forms. No difference was found in any of the mutants generated compared to the control.

**A)**

**B)**


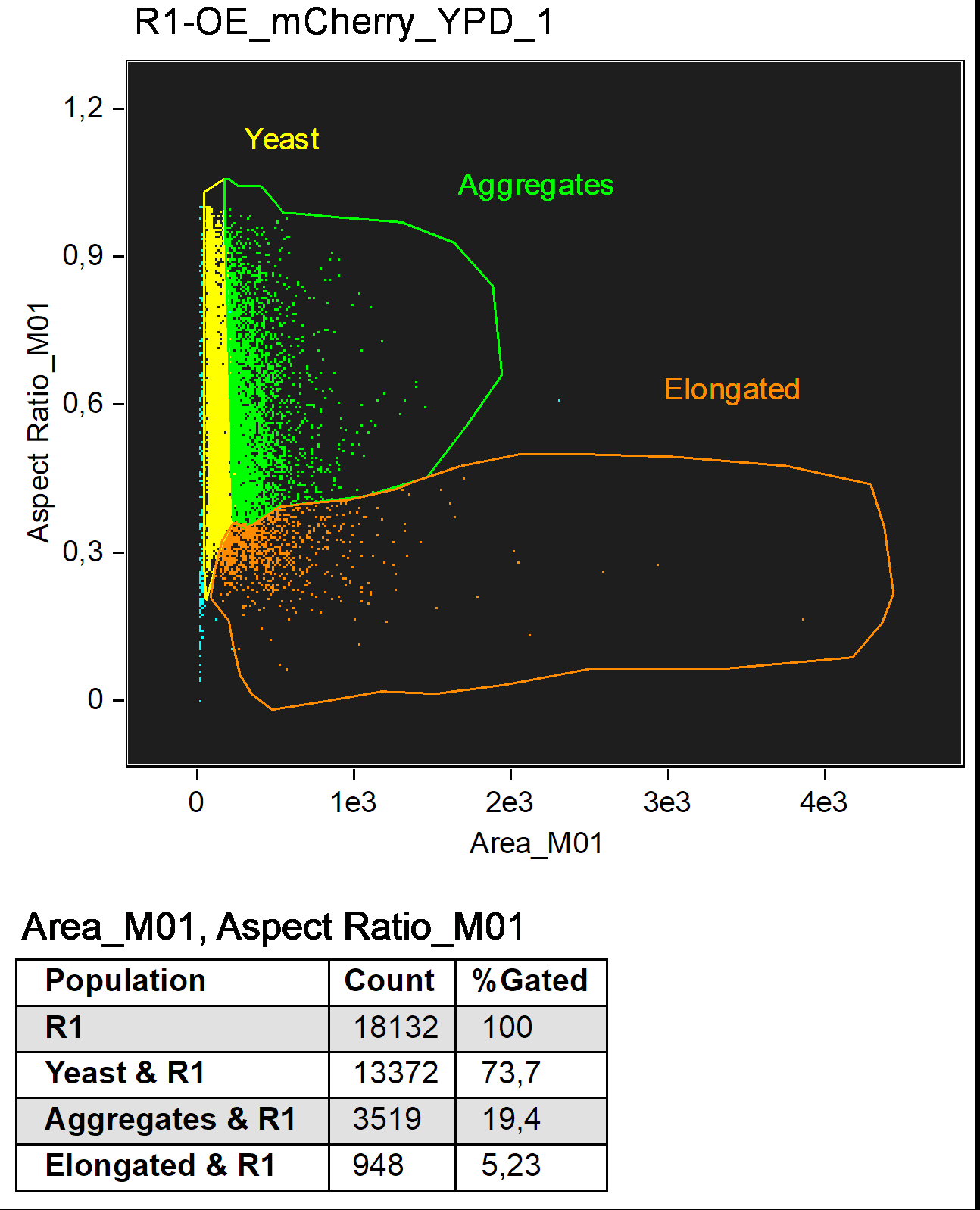

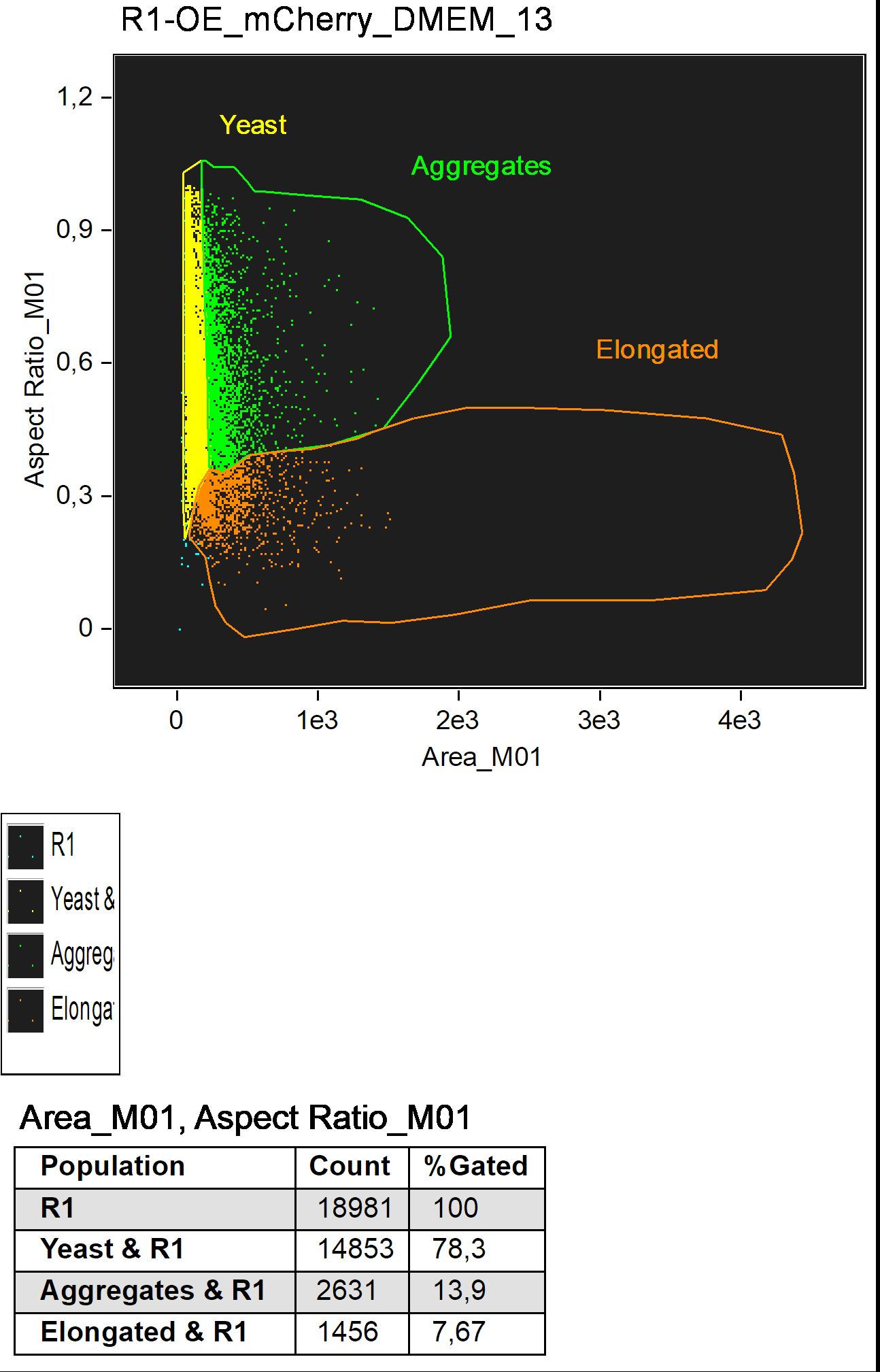

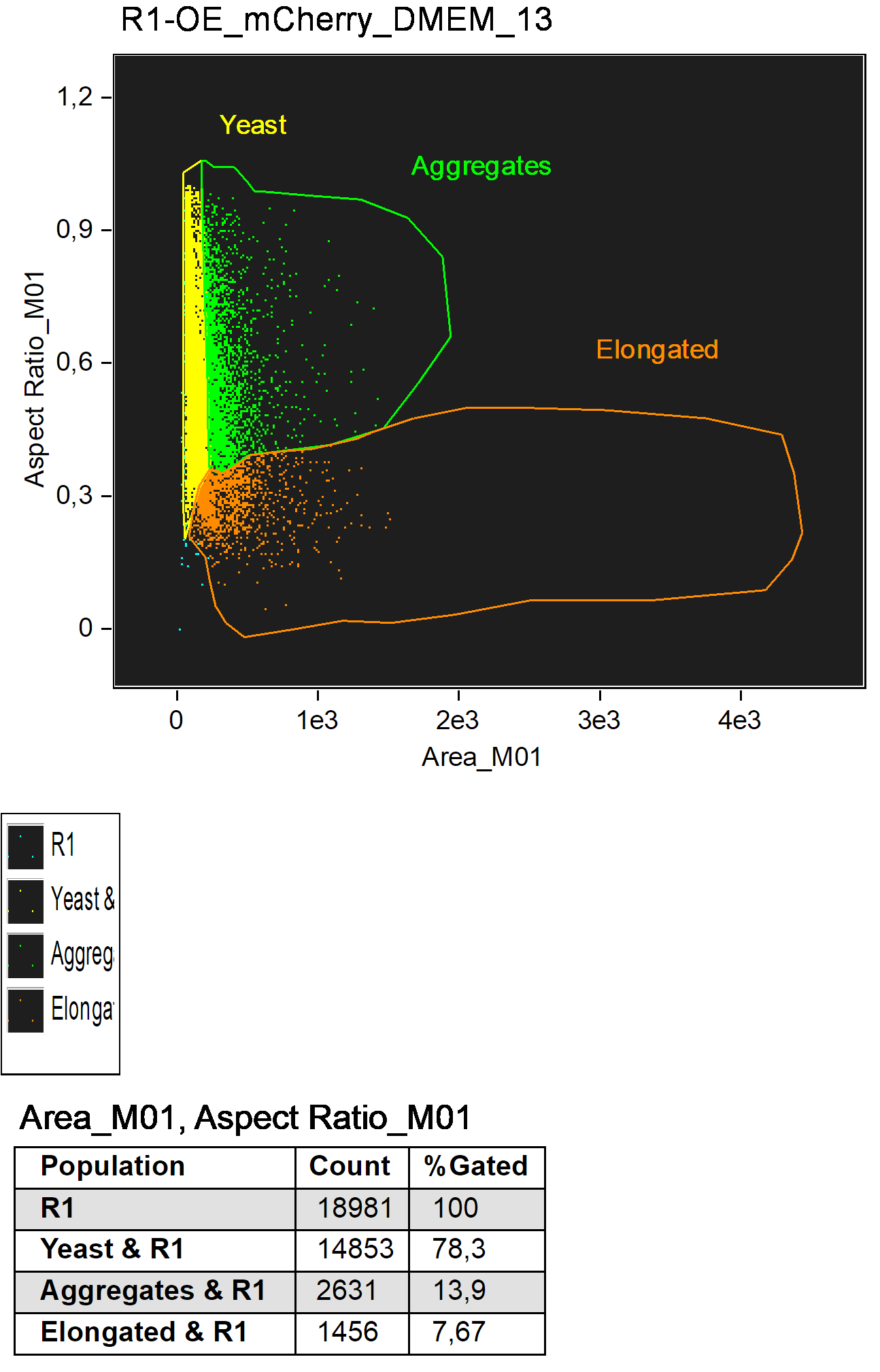

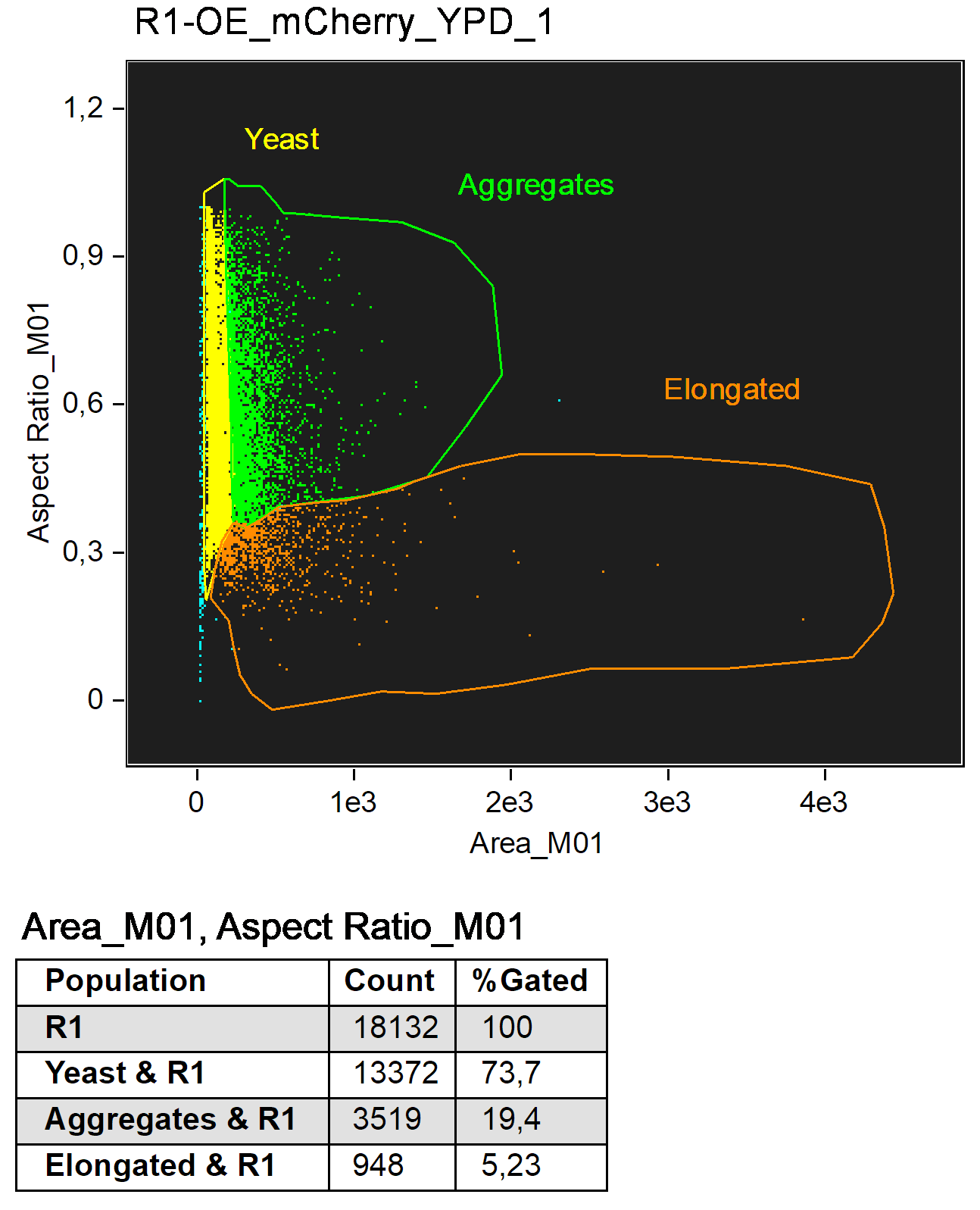


YPD medium

DMEM + 10% FBS medium


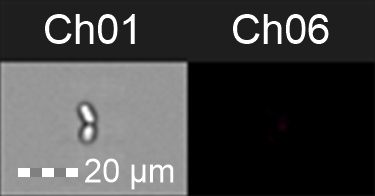

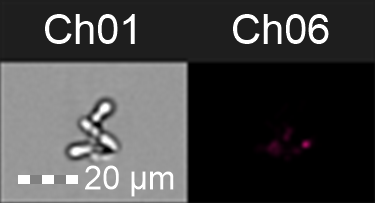

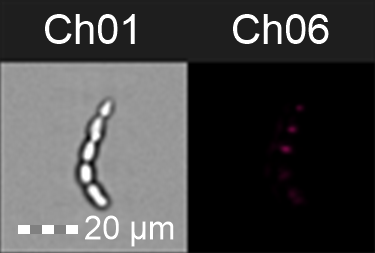

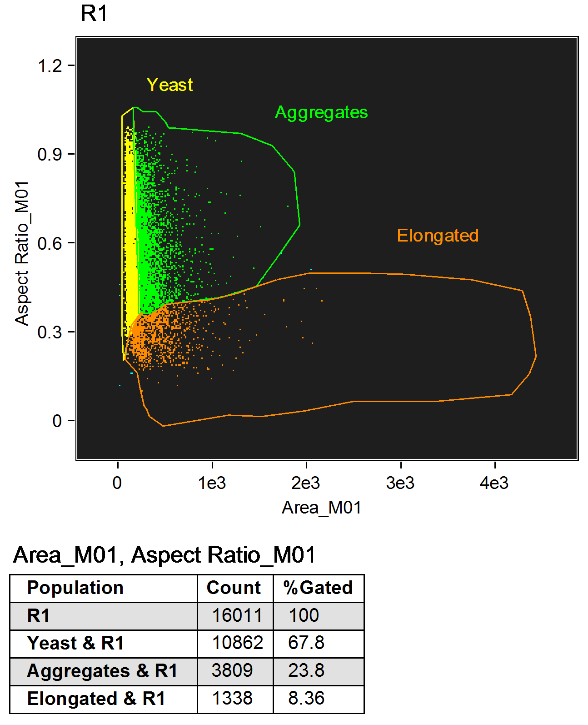

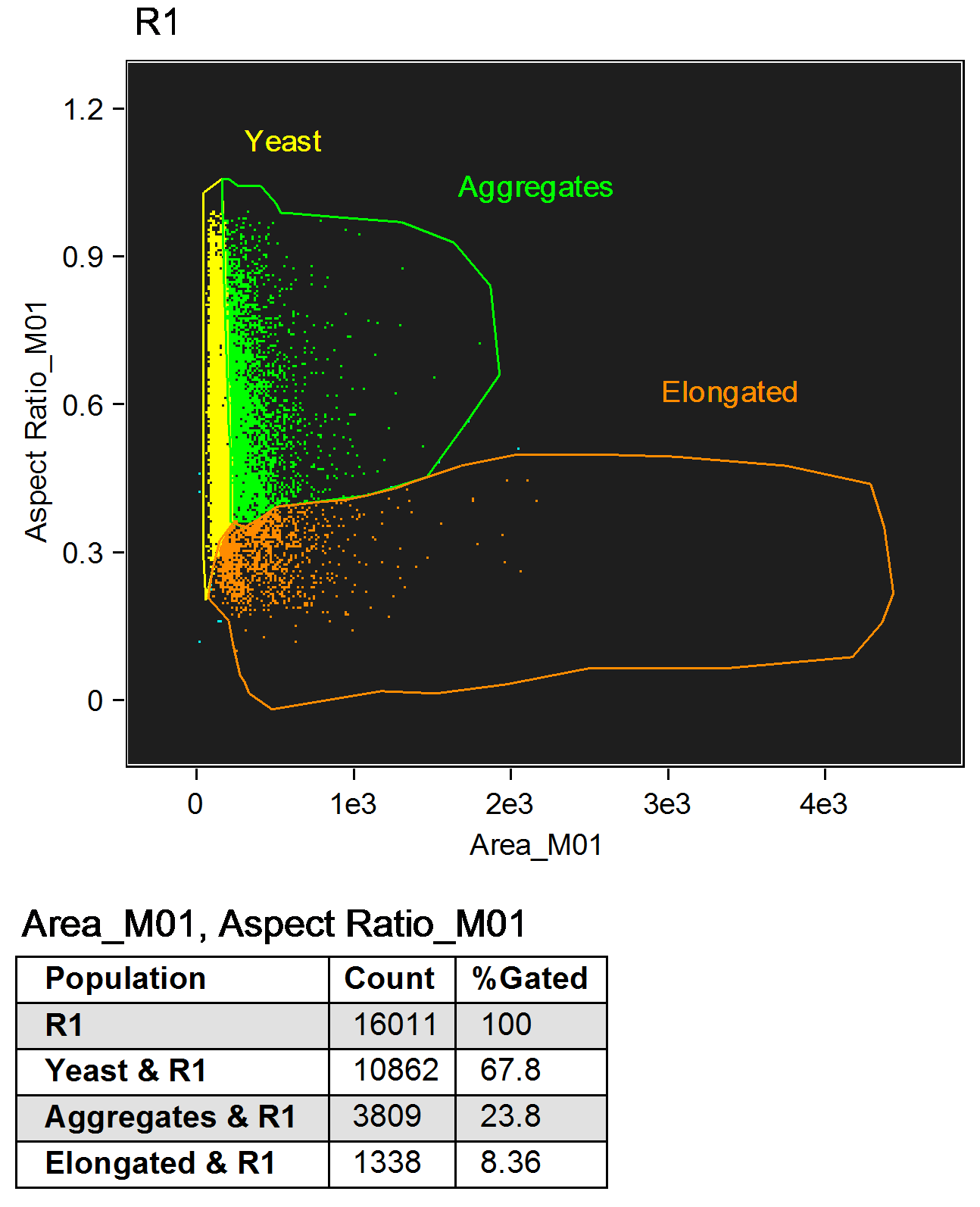

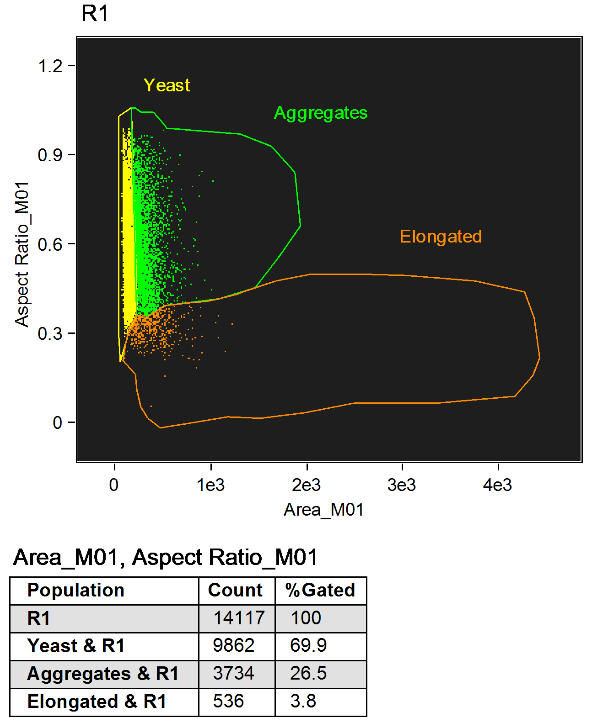

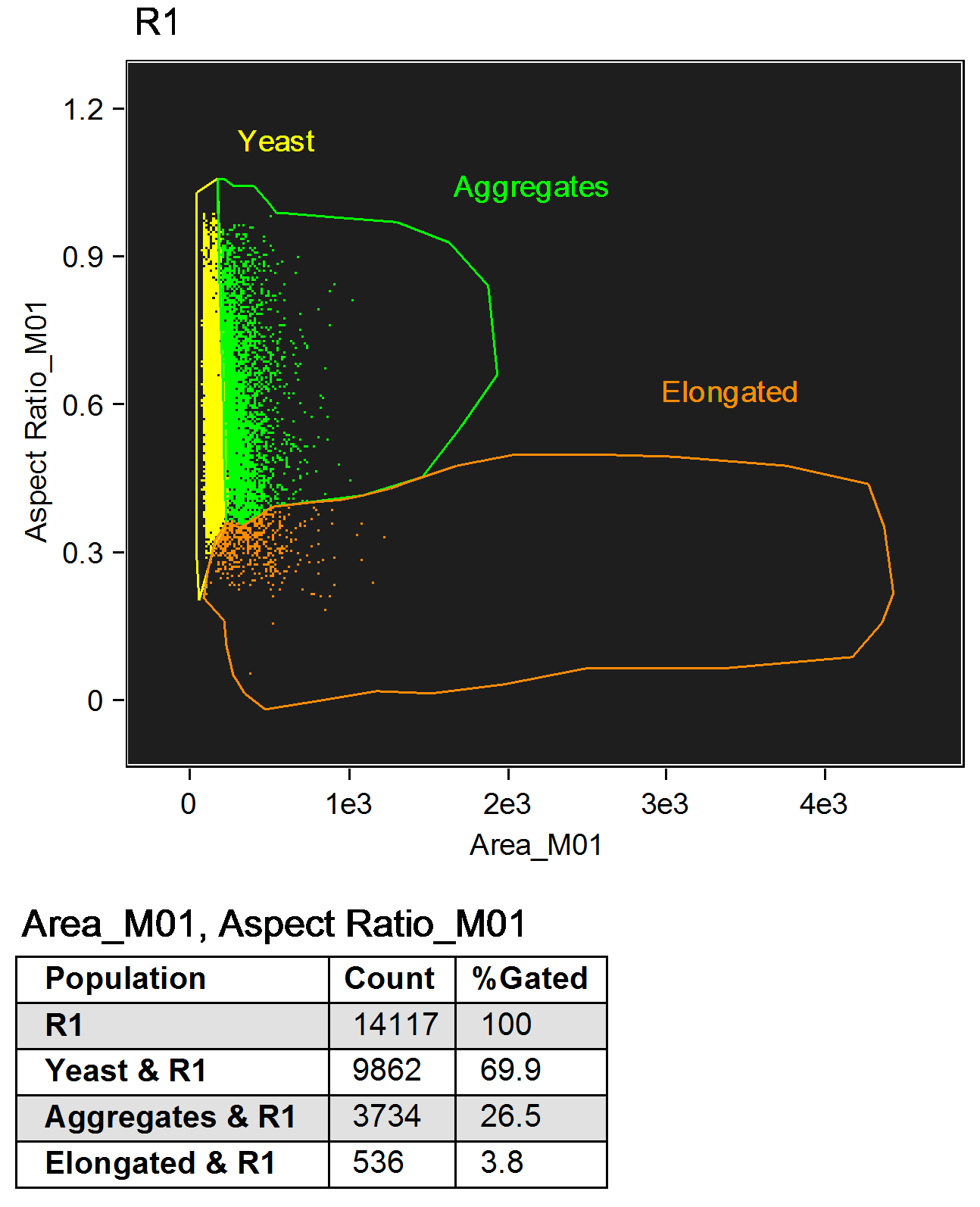


**C)**

**Figure S8:** **Biofilm forming capacity of the overexpression mutants.**

MTT assay was carried out according to the manufacturer’s instructions and OD_540_ values related to the OE strains were normalized to that of the CLIB214 control strain. N=3 with 8 parallel samples per experiment. Statistical analysis was performed with one-way ANOVA with Dunnett's multiple comparisons test. Only CPAR2_302400^OE^ showed significantly altered biofilm forming properties (****p<0.0001).

CPAR2_302400^OE^

**Normalized data**


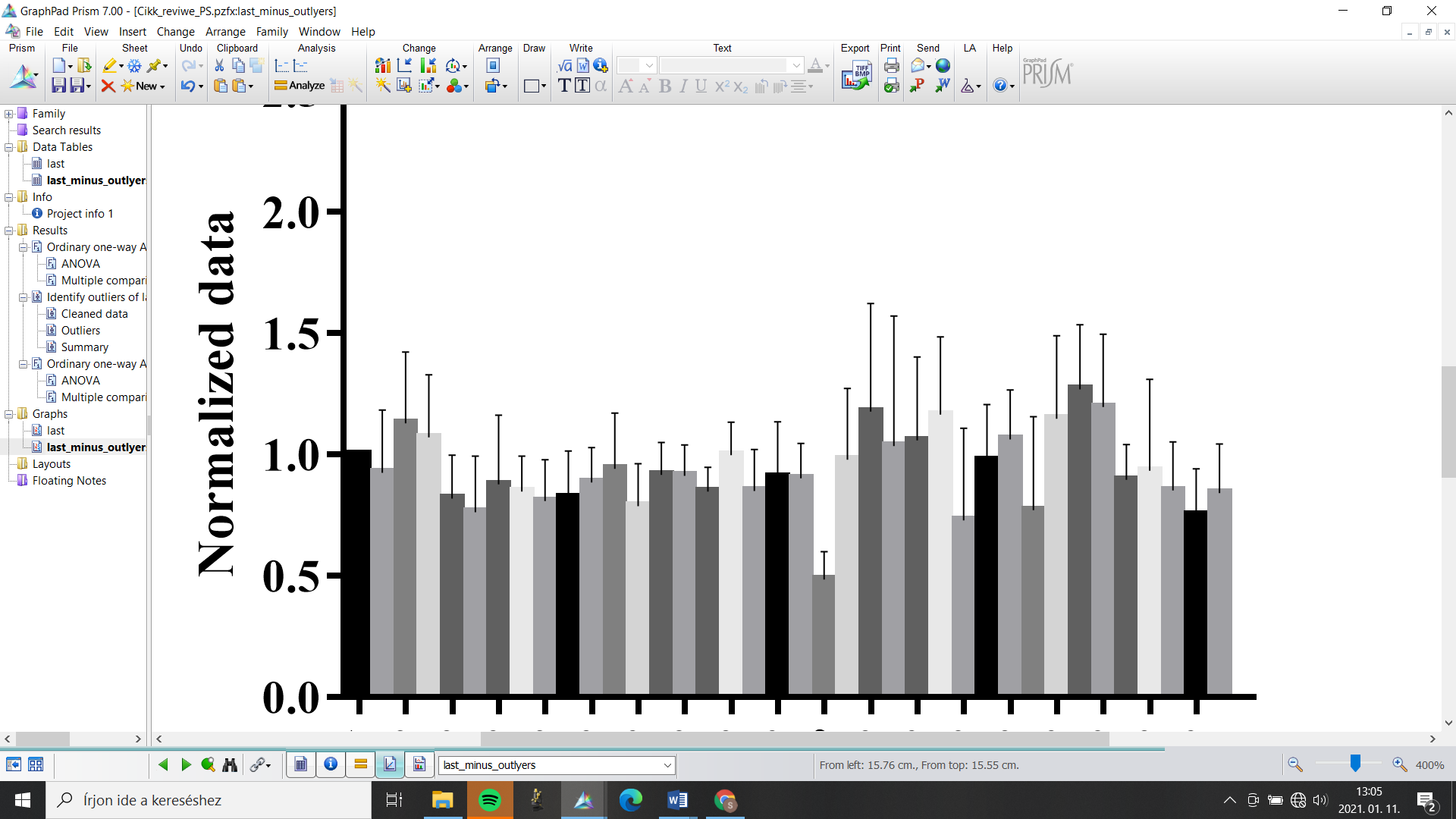


****

CPAR2_303240^OE^

CPAR2_303730^OE^

CPAR2_400270^OE^

CPAR2_406400^OE^

CPAR2_500180^OE^

CPAR2_500360^OE^

CPAR2_501400^OE^

CPAR2_503290^OE^

CPAR2_503760^OE^

CPAR2_602370^OE^

CPAR2_602820^OE^

CPAR2_602840^OE^

CPAR2_700550^OE^

CPAR2_703840^OE^

CPAR2_804030^OE^

CPAR2_805930^OE^

CPAR2_806950^OE^

**CLIB214**

CPAR2_100460^OE^

CPAR2_100470^OE^

CPAR2_100540^OE^

CPAR2_104420^OE^

CPAR2_105250^OE^

CPAR2_107020^OE^

CPAR2_107240^OE^

CPAR2_108840^OE^

CPAR2_109520^OE^

CPAR2_200040^OE^

CPAR2_200390^OE^

CPAR2_201920^OE^

CPAR2_204840^OE^

CPAR2_205060^OE^

CPAR2_208600^OE^

CPAR2_209240^OE^

CPAR2_209520^OE^

CPAR2_300080^OE^

CPAR2_301360^OE^

**2.0**

**1.5**

**1.0**

**0.5**

**0.0**

**Figure S9: Cell wall component analysis of the selected mutants.**

**A)** Representative figures present the chitin (Calcofluor white, CFW) and its oligomer (WGA-FITC) and alpha mannan (ConA-FITC) content of the CLIB214 and the CPAR2_107240^OE^ mutant strains. **B)** The figures give an example to the results of certain cell wall component analysis by flow cytometer (Ch-channel). No difference was found in any of the mutants generated compared to the control.

**A)**


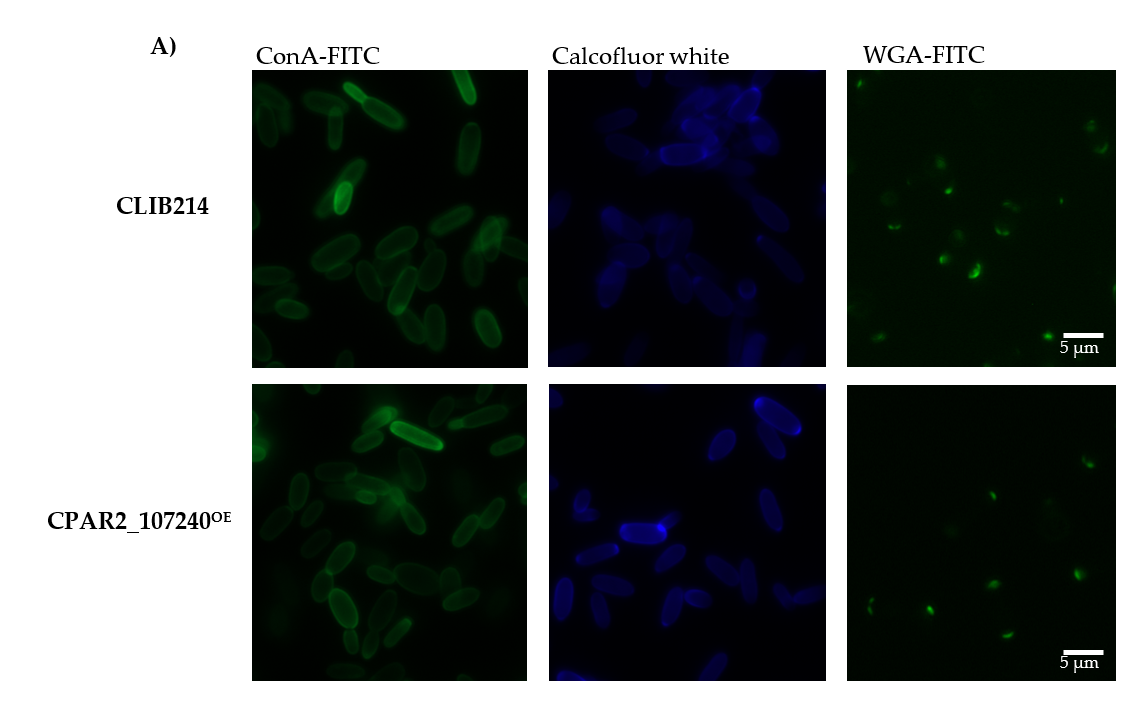


**CLIB214**

**CPAR2_107240^OE^**

**CPAR2_107240^OE^**

**CLIB214**

**B)**

**WGA-FITC**

**ConA-FITC**

**Calcofluor white**


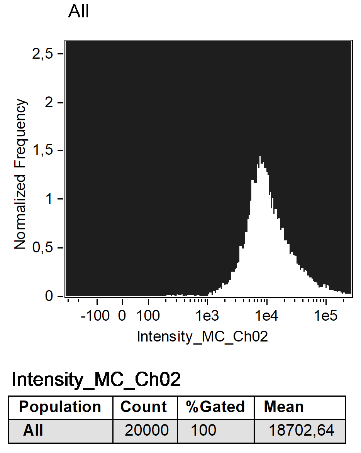

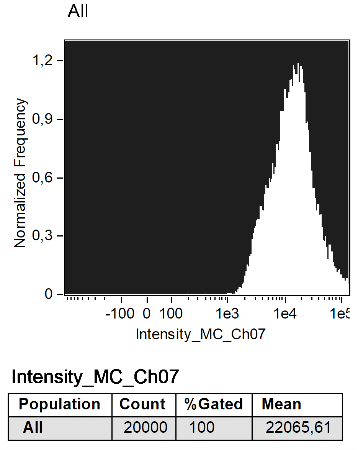

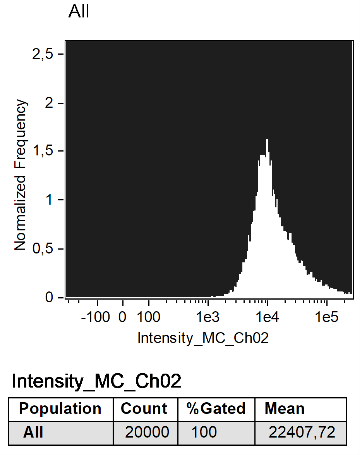

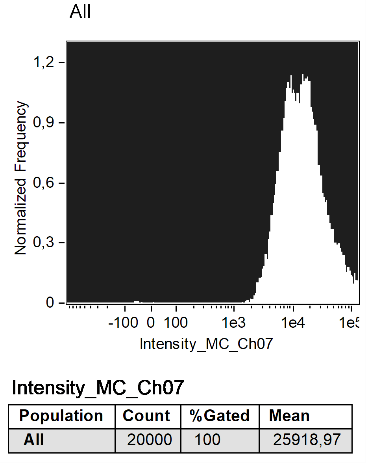

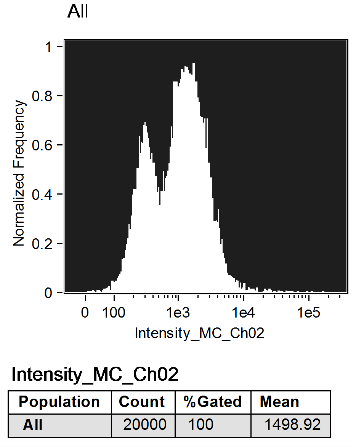

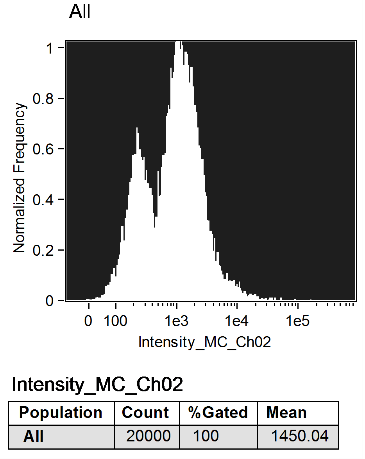


References

1. Laffey, S.F.; Butler, G. Phenotype switching affects biofilm formation by Candida parapsilosis. *Microbiology* **2005**, *151*, 1073–1081, doi:10.1099/mic.0.27739-0.
2. Holland, L.M.; Schröder, M.S.; Turner, S.A.; Taff, H.; Andes, D.; Grózer, Z.; Gácser, A.; Ames, L.; Haynes, K.; Higgins, D.G.; et al. Comparative Phenotypic Analysis of the Major Fungal Pathogens Candida parapsilosis and Candida albicans. *PLOS Pathog.* **2014**, *10*, e1004365.
3. Tóth, R.; Cabral, V.; Thuer, E.; Bohner, F.; Németh, T.; Papp, C.; Nimrichter, L.; Molnár, G.; Vágvölgyi, C.; Gabaldón, T.; et al. Investigation of Candida parapsilosis virulence regulatory factors during host-pathogen interaction. *Sci. Rep.* **2018**, *8*, 1346, doi:10.1038/s41598-018-19453-4.
4. Cillingová, A.; Zeman, I.; Tóth, R.; Neboháčová, M.; Dunčková, I.; Hölcová, M.; Jakúbková, M.; Gérecová, G.; Pryszcz, L.P.; Tomáška, Ľ.; et al. Eukaryotic transporters for hydroxyderivatives of benzoic acid. *Sci. Rep.* **2017**, *7*, 8998, doi:10.1038/s41598-017-09408-6.
5. Johnson, D.C.; Cano, K.E.; Kroger, E.C.; McNabb, D.S. Novel regulatory function for the CCAAT-binding factor in Candida albicans. *Eukaryot. Cell* **2005**, *4*, 1662–1676, doi:10.1128/EC.4.10.1662-1676.2005.
6. Shirayama, M.; Matsui, Y.; Tanaka, K.; Toh-e, A. Isolation of a CDC25 family gene, MSI2/LTE1, as a multicopy suppressor of ira1. *Yeast* **1994**, *10*, 451–461, doi:10.1002/yea.320100404.
7. Geymonat, M.; Spanos, A.; de Bettignies, G.; Sedgwick, S.G. Lte1 contributes to Bfa1 localization rather than stimulating nucleotide exchange by Tem1. *J. Cell Biol.* **2009**, *187*, 497–511, doi:10.1083/jcb.200905114.
8. Percival-Smith, A.; Segall, J. Characterization and mutational analysis of a cluster of three genes expressed preferentially during sporulation of Saccharomyces cerevisiae. *Mol. Cell. Biol.* **1986**, *6*, 2443–2451, doi:10.1128/mcb.6.7.2443.
9. Braun, B.R.; Johnson, A.D. Control of filament formation in Candida albicans by the transcriptional repressor TUP1. *Science* **1997**, *277*, 105–109, doi:10.1126/science.277.5322.105.
10. Laprade, L.; Boyartchuk, V.L.; Dietrich, W.F.; Winston, F. Spt3 plays opposite roles in filamentous growth in Saccharomyces cerevisiae and *Candida albicans* and is required for *C. albicans* virulence. *Genetics* **2002**, *161*, 509–519.
11. Liu, H.; Köhler, J.; Fink, G.R. Suppression of hyphal formation in Candida albicans by mutation of a STE12 homolog. *Science* **1994**, *266*, 1723–1726, doi:10.1126/science.7992058.
12. Nobile, C.J.; Bruno, V.M.; Richard, M.L.; Davis, D.A.; Mitchell, A.P. Genetic control of chlamydospore formation in Candida albicans. *Microbiology* **2003**, *149*, 3629–3637, doi:10.1099/mic.0.26640-0.
13. Cooper, A.J.; Friedberg, E.C. A putative second adenylate kinase-encoding gene from the yeast Saccharomyces cerevisiae. *Gene* **1992**, *114*, 145–148, doi:10.1016/0378-1119(92)90721-z.
14. Gu, Y.; Gordon, D.M.; Amutha, B.; Pain, D. A GTP:AMP phosphotransferase, Adk2p, in Saccharomyces cerevisiae. Role of the C terminus in protein folding/stabilization, thermal tolerance, and enzymatic activity. *J. Biol. Chem.* **2005**, *280*, 18604–18609, doi:10.1074/jbc.M500847200.
15. Xiao, W.; Derfler, B.; Chen, J.; Samson, L. Primary sequence and biological functions of a Saccharomyces cerevisiae O6-methylguanine/O4-methylthymine DNA repair methyltransferase gene. *EMBO J.* **1991**, *10*, 2179–2186.
16. Mortimer, R.K.; Contopoulou, C.R.; King, J.S. Genetic and physical maps of Saccharomyces cerevisiae, Edition 11. *Yeast* **1992**, *8*, 817–902, doi:10.1002/yea.320081002.
17. Rothé, B.; Saliou, J.-M.; Quinternet, M.; Back, R.; Tiotiu, D.; Jacquemin, C.; Loegler, C.; Schlotter, F.; Peña, V.; Eckert, K.; et al. Protein Hit1, a novel box C/D snoRNP assembly factor, controls cellular concentration of the scaffolding protein Rsa1 by direct interaction. *Nucleic Acids Res.* **2014**, *42*, 10731–10747, doi:10.1093/nar/gku612.
18. Jones, D.G.L.; Rosamond, J. Isolation of a novel protein kinase-encoding gene from yeast by oligodeoxyribonucleotide probing. *Gene* **1990**, *90*, 87–92, doi:https://doi.org/10.1016/0378-1119(90)90442-T.
19. Mora-Montes, H.M.; Bates, S.; Netea, M.G.; Díaz-Jiménez, D.F.; López-Romero, E.; Zinker, S.; Ponce-Noyola, P.; Kullberg, B.J.; Brown, A.J.P.; Odds, F.C.; et al. Endoplasmic reticulum alpha-glycosidases of Candida albicans are required for N glycosylation, cell wall integrity, and normal host-fungus interaction. *Eukaryot. Cell* **2007**, *6*, 2184–2193, doi:10.1128/EC.00350-07.
20. Erbs, P.; Exinger, F.; Jund, R. Characterization of the Saccharomyces cerevisiae FCY1 gene encoding cytosine deaminase and its homologue FCA1 of Candida albicans. *Curr. Genet.* **1997**, *31*, 1–6, doi:10.1007/s002940050169.
21. Schweizer, A.; Rupp, S.; Taylor, B.N.; Röllinghoff, M.; Schröppel, K. The TEA/ATTS transcription factor CaTec1p regulates hyphal development and virulence in Candida albicans. *Mol. Microbiol.* **2000**, *38*, 435–445, doi:10.1046/j.1365-2958.2000.02132.x.
22. Miyakawa, Y. Identification of a Candida albicans homologue of the PHO85 gene, a negative regulator of the PHO system in Saccharomyces cerevisiae. *Yeast* **2000**, *16*, 1045–1051, doi:10.1002/1097-0061(200008)16:11<1045::AID-YEA595>3.0.CO;2-L.
23. Doedt, T.; Krishnamurthy, S.; Bockmühl, D.P.; Tebarth, B.; Stempel, C.; Russell, C.L.; Brown, A.J.P.; Ernst, J.F. APSES proteins regulate morphogenesis and metabolism in Candida albicans. *Mol. Biol. Cell* **2004**, *15*, 3167–3180, doi:10.1091/mbc.e03-11-0782.
24. Kruppa, M.; Jabra-Rizk, M.A.; Meiller, T.F.; Calderone, R. The histidine kinases of Candida albicans: regulation of cell wall mannan biosynthesis. *FEMS Yeast Res.* **2004**, *4*, 409–416, doi:10.1016/S1567-1356(03)00201-0.
25. Romero, P.A.; Lussier, M.; Véronneau, S.; Sdicu, A.M.; Herscovics, A.; Bussey, H. Mnt2p and Mnt3p of Saccharomyces cerevisiae are members of the Mnn1p family of alpha-1,3-mannosyltransferases responsible for adding the terminal mannose residues of O-linked oligosaccharides. *Glycobiology* **1999**, *9*, 1045–1051, doi:10.1093/glycob/9.10.1045.
26. Bates, S.; Hall, R.A.; Cheetham, J.; Netea, M.G.; MacCallum, D.M.; Brown, A.J.P.; Odds, F.C.; Gow, N.A.R. Role of the Candida albicans MNN1 gene family in cell wall structure and virulence. *BMC Res. Notes* **2013**, *6*, 294, doi:10.1186/1756-0500-6-294.
27. Sherlock, G.; Bahman, A.M.; Mahal, A.; Shieh, J.C.; Ferreira, M.; Rosamond, J. Molecular cloning and analysis of CDC28 and cyclin homologues from the human fungal pathogen Candida albicans. *Mol. Gen. Genet.* **1994**, *245*, 716–723, doi:10.1007/BF00297278.
28. Swoboda, R.K.; Bertram, G.; Hollander, H.; Greenspan, D.; Greenspan, J.S.; Gow, N.A.; Gooday, G.W.; Brown, A.J. Glycolytic enzymes of Candida albicans are nonubiquitous immunogens during candidiasis. *Infect. Immun.* **1993**, *61*, 4263–4271, doi:10.1128/IAI.61.10.4263-4271.1993.
29. Yan, L.; Zhang, J.-D.; Cao, Y.-B.; Gao, P.-H.; Jiang, Y.-Y. Proteomic analysis reveals a metabolism shift in a laboratory fluconazole-resistant Candida albicans strain. *J. Proteome Res.* **2007**, *6*, 2248–2256, doi:10.1021/pr060656c.
30. Bates, S.; MacCallum, D.M.; Bertram, G.; Munro, C.A.; Hughes, H.B.; Buurman, E.T.; Brown, A.J.P.; Odds, F.C.; Gow, N.A.R. Candida albicans Pmr1p, a secretory pathway P-type Ca2+/Mn2+-ATPase, is required for glycosylation and virulence. *J. Biol. Chem.* **2005**, *280*, 23408–23415, doi:10.1074/jbc.M502162200.
31. Mitchell, D.A.; Vasudevan, A.; Linder, M.E.; Deschenes, R.J. Protein palmitoylation by a family of DHHC protein S-acyltransferases. *J. Lipid Res.* **2006**, *47*, 1118–1127, doi:10.1194/jlr.R600007-JLR200.
32. Mortimer, R.K.; Hawthorne, D.C. Genetic mapping in Saccharomyces. *Genetics* **1966**, *53*, 165–173.
33. Mannhaupt, G.; Pohlenz, H.D.; Seefluth, A.K.; Pilz, U.; Feldmann, H. Yeast homoserine kinase. Characteristics of the corresponding gene, THR1, and the purified enzyme, and evolutionary relationships with other enzymes of threonine metabolism. *Eur. J. Biochem.* **1990**, *191*, 115–122, doi:10.1111/j.1432-1033.1990.tb19100.x.
34. Nogi, Y.; Yano, R.; Dodd, J.; Carles, C.; Nomura, M. Gene RRN4 in Saccharomyces cerevisiae encodes the A12.2 subunit of RNA polymerase I and is essential only at high temperatures. *Mol. Cell. Biol.* **1993**, *13*, 114–122, doi:10.1128/MCB.13.1.114.
35. Yoshihisa, T.; Anraku, Y. Nucleotide sequence of AMS1, the structure gene of vacuolar alpha-mannosidase of Saccharomyces cerevisiae. *Biochem. Biophys. Res. Commun.* **1989**, *163*, 908–915, doi:10.1016/0006-291x(89)92308-5.
36. Yoshihisa, T.; Anraku, Y. A novel pathway of import of alpha-mannosidase, a marker enzyme of vacuolar membrane, in Saccharomyces cerevisiae. *J. Biol. Chem.* **1990**, *265*, 22418–22425.
37. Shi, Q.-M.; Wang, Y.-M.; Zheng, X.-D.; Lee, R.T.H.; Wang, Y. Critical role of DNA checkpoints in mediating genotoxic-stress-induced filamentous growth in Candida albicans. *Mol. Biol. Cell* **2007**, *18*, 815–826, doi:10.1091/mbc.e06-05-0442.
38. Fleischer, T.C.; Weaver, C.M.; McAfee, K.J.; Jennings, J.L.; Link, A.J. Systematic identification and functional screens of uncharacterized proteins associated with eukaryotic ribosomal complexes. *Genes Dev.* **2006**, *20*, 1294–1307, doi:10.1101/gad.1422006.
39. Prakash, L. Effect of Genes Controlling Radiation Sensitivity on Chemically Induced Mutations in *Saccharomyces cerevisiae*. *Genetics* **1976**, *83*, 285–301.
40. Bailly, V.; Lauder, S.; Prakash, S.; Prakash, L. Yeast DNA repair proteins Rad6 and Rad18 form a heterodimer that has ubiquitin conjugating, DNA binding, and ATP hydrolytic activities. *J. Biol. Chem.* **1997**, *272*, 23360–23365, doi:10.1074/jbc.272.37.23360.
41. Feaver, W.J.; Henry, N.L.; Wang, Z.; Wu, X.; Svejstrup, J.Q.; Bushnell, D.A.; Friedberg, E.C.; Kornberg, R.D. Genes for Tfb2, Tfb3, and Tfb4 subunits of yeast transcription/repair factor IIH. Homology to human cyclin-dependent kinase activating kinase and IIH subunits. *J. Biol. Chem.* **1997**, *272*, 19319–19327, doi:10.1074/jbc.272.31.19319.
42. Cairns, B.R.; Lorch, Y.; Li, Y.; Zhang, M.; Lacomis, L.; Erdjument-Bromage, H.; Tempst, P.; Du, J.; Laurent, B.; Kornberg, R.D. RSC, an essential, abundant chromatin-remodeling complex. *Cell* **1996**, *87*, 1249–1260, doi:10.1016/s0092-8674(00)81820-6.
43. Landrieu, I.; Vandenbol, M.; Härtlein, M.; Portetelle, D. Mitochondrial asparaginyl-tRNA synthetase is encoded by the yeast nuclear gene YCR24c. *Eur. J. Biochem.* **1997**, *243*, 268–273, doi:10.1111/j.1432-1033.1997.0268a.x.
44. Audhya, A.; Loewith, R.; Parsons, A.B.; Gao, L.; Tabuchi, M.; Zhou, H.; Boone, C.; Hall, M.N.; Emr, S.D. Genome-wide lethality screen identifies new PI4,5P2 effectors that regulate the actin cytoskeleton. *EMBO J.* **2004**, *23*, 3747–3757, doi:10.1038/sj.emboj.7600384.
45. Inoue, S.B.; Takewaki, N.; Takasuka, T.; Mio, T.; Adachi, M.; Fujii, Y.; Miyamoto, C.; Arisawa, M.; Furuichi, Y.; Watanabe, T. Characterization and gene cloning of 1,3-beta-D-glucan synthase from Saccharomyces cerevisiae. *Eur. J. Biochem.* **1995**, *231*, 845–854, doi:10.1111/j.1432-1033.1995.tb20770.x.
46. Tone, Y.; Tanahashi, N.; Tanaka, K.; Fujimuro, M.; Yokosawa, H.; Toh-e, A. Nob1p, a new essential protein, associates with the 26S proteasome of growing Saccharomyces cerevisiae cells. *Gene* **2000**, *243*, 37–45, doi:10.1016/s0378-1119(99)00566-1.
47. Prelich, G. Gene overexpression: uses, mechanisms, and interpretation. *Genetics* **2012**, *190*, 841–854, doi:10.1534/genetics.111.136911.
48. Németh, T.; Papp, C.; Vagvolgyi, C.; Chakraborty, T.; Gacser, A. Identification and Characterization of a Neutral Locus for Knock-in Purposes in C. parapsilosis. *Front. Microbiol.* **2020**, *11*, 1194.
