# Supplementary material for: A Candida parapsilosis Overexpression Collection Reveals Genes Required for Pathogenesis
Source: J Fungi (Basel). 2021 Jan 29;7(2):97. doi: 10.3390/jof7020097 (PMC7911391; doi:10.3390/jof7020097)
Supplement: Supplementary file 1 [file jof-07-00097-s001.zip › Table_S2.pdf]

Table S2: Applied stressors and their concentrations in solid YPD media for spot assay analysis.

**pH**
YPD pH 4, 5, 6, 7, 8 (McIlvaine buffers)<br>(0.2 M Na_2HPO_4 and 0.1 M Citric acid)
**Osmotic stress**
YPD + 3% w/V Glycerol<br>YPD + 1 M NaCl<br>YPD + 1 M Sorbitol
**Oxidative and metal ion stress**
YPD + 2 mM $CuCl_2$<br>YPD + 2 mM $CuSO_4$<br>YPD + 12.5 µM $CdSO_4$<br>YPD + 5 mM H_2O_2
**Cell wall perturbants**
YPD + 10 mM Caffeine<br>YPD + 10, 25, 50 µg/ml Calcofluor White (CFW)<br>YPD + 10, 25, 50, 75, 100 µg/ml Congo Red (CR)
**Membrane perturbant**
YPD + 0.04% w/V SDS
**Metal ion starvation**
YPD + 0.25 mM EDTA<br>YPD + 0.15 mM BPS<br>YPD + 0.5 mM BPS + 2 µM Hemin
